# Spectroscopic Data for the Rapid Assessment of Microbiological Quality of Chicken Burgers

**DOI:** 10.3390/foods11162386

**Published:** 2022-08-09

**Authors:** Lemonia-Christina Fengou, Yunge Liu, Danai Roumani, Panagiotis Tsakanikas, George-John E. Nychas

**Affiliations:** 1 Laboratory of Microbiology and Biotechnology of Foods, Department of Food Science and Human Nutrition, School of Food and Nutritional Sciences, Agricultural University of Athens, Iera Odos 75, 11855 Athens, Greece; 2 Laboratory of Beef Processing and Quality Control, College of Food Science and Engineering, Shandong Agricultural University, Tai’an 271018, China

**Keywords:** chicken burgers, Fourier transform infrared (FTIR) spectroscopy, multispectral imaging (MSI), machine learning

## Abstract

The rapid assessment of the microbiological quality of highly perishable food commodities is of great importance. Spectroscopic data coupled with machine learning methods have been investigated intensively in recent years, because of their rapid, non-destructive, eco-friendly qualities and their potential to be used on-, in- or at-line. In the present study, the microbiological quality of chicken burgers was evaluated using Fourier transform infrared (FTIR) spectroscopy and multispectral imaging (MSI) in tandem with machine learning algorithms. Six independent batches were purchased from a food industry and stored at 0, 4, and 8 °C. At regular time intervals (specifically every 24 h), duplicate samples were subjected to microbiological analysis, FTIR measurements, and MSI sampling. The samples (n = 274) acquired during the data collection were classified into three microbiological quality groups: “satisfactory”: 4–7 log CFU/g, “acceptable”: 7–8 log CFU/g, and “unacceptable”: >8 logCFU/g. Subsequently, classification models were trained and tested (external validation) with several machine learning approaches, namely partial least squares discriminant analysis (PLSDA), support vector machine (SVM), random forest (RF), logistic regression (LR), and ordinal logistic regression (OLR). Accuracy scores were attained for the external validation, exhibiting FTIR data values in the range of 79.41–89.71%, and, for the MSI data, in the range of 74.63–85.07%. The performance of the models showed merit in terms of the microbiological quality assessment of chicken burgers.

## 1. Introduction

According to the FAO [1], in 2020, poultry meat was the most produced meat type (133.3 million tonnes) in the world, relative to pig (109.2 million tonnes), bovine (71.4 million tonnes), and ovine (16 million tonnes) meat. The rapid quality assessment of this perishable and widely consumed food commodity is of great importance for assuring food quality throughout the supply chain and minimizing concurrent food waste. The current approach to quality assessment relies heavily on time-consuming, sample destructive and costly methods, for example, microbiological analyses (e.g., plate count method) and molecular methods (e.g., real-time polymerase chain reaction), which, most often, are applied only to the end-product. In contrast, methods based on spectral, or imaging, acquisition can provide a large amount of data in a very short time. Data mining is of crucial importance to extract useful information from these data with respect to the efficiency of models used. The application of these methods on a large scale would enable the rapid detection of deterioration in quality, the on-time implementation of corrective actions and quality assurance of the food commodities examined [2].

Spectroscopic methods coupled with machine learning algorithms have been intensively investigated, especially in the case of red meat [3,4,5], but also for poultry meat [6,7] for quality assessment and detection of food fraud. Poultry meat has been investigated using several quality parameters, such as microbial spoilage, pH, sensory evaluation, water holding capacity, and color [8,9], in parallel to the acquisition of spectral data, such as near infrared (NIR), Fourier transform infrared (FTIR), Raman, visible-near infrared (Vis-NIR), and hyper- and multi-spectral imaging (HSI, MSI) for the rapid assessment of quality [10,11,12,13].

FTIR and MSI allow for screening of samples in a very short time and can be used non-destructively, while assessing many predictor features. Vibrational spectroscopy utilizes the interaction between chemical bonds of a given compound with infrared radiation. This produces an infrared spectrum which represents a fingerprint for the analyzed sample [14]. MSI is also a widely used technique, providing both spatial and spectral information. Spectrometers analyze only a small portion of the food sample (point measurement); therefore, the spectra are not representative of the whole sample. On the other hand, imaging approaches consider the whole sample, or at least a large part of it, and provide much more representative and detailed measurements. Apart from spectral information, MSI also provides spatial information and, more importantly, enables the representation of the whole sample instead of a few sites/points only [15,16]. In essence, MSI data corresponds to a three-dimensional (3D) image cube, holding both spatial and spectral information, while FTIR data is a vector with features typically in the region of 4000–400 cm^−1^. Spectroscopic techniques are not able to directly detect microorganisms, but changes related to the metabolic activity of microorganisms (i.e., by-products) are captured by FTIR [17,18,19,20]. Changes related to respiratory pigments (e.g., myoglobin, oxymyoglobin, metmyoglobin) and proteins, and to moisture content can be captured by MSI in the region of VIS-NIR [21,22,23,24]. This acquired information, after appropriate pre-processing and data manipulation, is fed into machine learning algorithms to reveal the hidden “correlations” of spectral-related data to microbial quality measured using “gold standard” microbiological approaches in a wet laboratory. Thus, data collection is usually followed by several data handling processes, such as data cleaning, feature selection, model development and evaluation.

In general, sensor-based acquired data, either spectral or spectral-imaging data, are handled using various machine learning approaches to extract and select the most informative features for the development of robust and generalized models able to efficiently predict quality parameters. In this framework, partial least squares (PLS), principal component analysis (PCA) and k-nearest neighbor (kNN) approaches have been applied on spectroscopic data for food quality, safety, and fraud assessment purposes, while, additionally, machine learning methods have also been applied, such as decision tree (DT), support vector machine (SVM), naïve Bayes (NB), random forest (RF), and logistic regression (LR) approaches [25,26,27,28].

It is of note that chicken breast fillets [29,30] are the most common tissue used in related studies compared to other chicken meat tissues or products [31]. The wide applicability, and the rapid and simple application of these sampling approaches, has previously been demonstrated, but certain limitations have also been apparent. For example, the need for large and diverse datasets, simulating real life situations as closely as possible (e.g., different conditions: storage time and temperatures, sample treatment, variability within- and between-batches), has been highlighted, not only in food-related domains, but in all domains where machine learning is employed, in order for robust models to be developed (see e.g., Tsakanikas [32]). Moreover, the product-specific character of data collected constrains the large-scale and universal application of these methods and reinforces the use of approaches, which are case-, product-, sensor-, or algorithm-specific [15].

Chicken burgers are a highly perishable food commodity subject to high consumer demand. Some of the intrinsic factors affecting food quality changes during shelf life include the ingredients, the pH, and the microflora, while extrinsic factors also play an important part, such as processing methods, storage conditions, the packaging used and the conditions during distribution [33,34]. The variability in recipes used, and industry practices, in combination with large-scale production, set the final product highly industry-specific. The assessment of the microbiological quality of poultry products, such as chicken burgers, has not been investigated, at least, to our knowledge, apart from in [31], where MSI data were employed for the determination of “time from slaughter”. In the present study, the aim was to predict the microbiological quality of the product, with FTIR data collected in parallel. The focus of the research was on investigating whether the rapid quality assessment of a commercial poultry product (i.e., a mixture of chicken meat, bread, vegetables, and flavorings) was feasible. For this purpose, different machine learning algorithms (PLSDA, SVM, RF, LR, OLR) were applied on data from two different sensors (FTIR, MSI). The samples were collected from six independent batches, purchased in 2018 and 2020, and were stored at three different temperatures (0, 4, 8 °C).

## 2. Materials and Methods

### 2.1. Purchase and Storage Conditions of Chicken Burgers

A commercial poultry product (i.e., chicken burgers) was provided to the laboratory by the food industry company KOTINO S.A., located in Athens, Greece. The chicken burgers consisted of whole boned chicken (76%), fresh onions, fresh peppers, soy vegetable protein, flavorings (mustard, pepper, curry), dried breadcrumb, sunflower oil, salt, yeast extract, and sodium caseinate. Two burgers (ca. 100 g each) were packaged in each styrofoam tray and wrapped with cling film. Subsequently, the samples were stored at 0, 4 and 8 °C in high-precision (±0.5 °C) programmable incubators (MIR-153, Sanyo Electric Co., Osaka, Japan) in dark conditions. Six chronically independent batches (b1, b2, b3, b4, b5, b6) were purchased (i.e., experimental replicates); two of which were purchased as fresh (year of purchase 2018) and four as frozen (year of purchase 2020). In the last case the samples were thawed under refrigeration conditions (0 or 4 °C) for 24–48 h, as recommended by [35] for safe food handling. The samples were analyzed every 24 h during storage. At every sampling point, duplicate chicken burgers (i.e., sample replicates) were subjected to: (i) microbiological analyses and pH measurements, (ii) Fourier transform infrared (FTIR) spectroscopy measurements, and (iii) multispectral imaging (MSI) acquisition. In total, 274 samples were analyzed.

### 2.2. Microbiological Analysis and pH Measurement

Chicken burger samples (25 g) were weighed aseptically, 225 mL of sterile quarter strength Ringer’s solution were added (Lab M Limited, Lancashire, UK) in a Stomacher bag (400-mL sterile, Seward Medical, London, UK), and the mixture homogenized in a Stomacher apparatus (Lab Blender 400, Seward Medical, London, UK) for 60 s at room temperature. Subsequently, serial decimal dilutions were prepared, 0.1 mL of appropriate decimal dilution was spread on the media, tryptic glucose yeast agar (Plate Count Agar; Biolife, Milan, Italy), which was then incubated at 25 °C for 72 h, for the enumeration of total mesophiles (total viable counts, TVC).

The samples from b1 and b2 were subjected to microbiological analysis for microbial groups *Pseudomonas* spp., *Brochothrix thermosphacta*, lactic acid bacteria (LAB), and Enterobacteriaceae at every sampling point (every 24 h); the samples were also analyzed for yeasts and molds every 72 h. The samples from b3-b6 were analyzed for the above microbial groups at only some sampling points. A spread method was applied for the enumeration of *Pseudomonas* spp. on the substrate of *Pseudomonas* agar base with the selective supplement cephalothin-fucidin-cetrimide (Lab M Limited, Lancashire, UK), and incubated at 25 °C for 48 h; for *B. thermosphacta* on streptomycin thallous acetate-actidione agar (STAA, Biolife, Milan, Italy), incubated at 25 °C for 48 h; and for molds and yeasts on Rose Bengal chloramphenicol agar (RBC, Lab M Limited, Lancashire, UK), incubated at 25 °C for 5 days. Furthermore, 1 mL of appropriate serial decimal dilutions of the chicken burger homogenates was pour-plated in the following media: de Man, Rogosa and Sharpe agar (MRS, Biolife, Milan, Italy) for the enumeration of LAB incubated at 30 °C for 72 h; and violet red bile glucose agar (VRBG, Biolife, Milan, Italy) for Enterobacteriaceae, incubated at 37 °C for 24 h. The microbial colonies were enumerated, and the microbiological data were converted to log CFU/g.

The pH values were recorded from the homogenate of chicken burger (1:10) after microbiological analysis [17] using a digital pH meter (RL150, Russell pH, Cork, Ireland) with a glass electrode (Metrohm AG, Herisau, Switzerland).

### 2.3. Spectroscopy-Based Sensors

#### 2.3.1. FTIR Measurements

An FTIR-6200 JASCO spectrometer (Jasco Corp., Tokyo, Japan) and a ZnSe 45° HATR (horizontal attenuated total reflectance) crystal (PIKE Technologies, Madison, Wisconsin, USA) were used for the collection of FTIR spectral data. In total, 274 spectra were collected over the wavenumber range of 4000 to 400 cm^−1^, using the Spectra Manager™ Code of Federal Regulations (CFR) software version 2 (Jasco Corp., Tokyo, Japan) by accumulating 100 scans. The process is described in more detail in Argyri et al. [36]. The FTIR spectra used for further analyses were in the wavenumber range of 1800–900 cm^−1^.

#### 2.3.2. MSI Acquisition

The VideometerLab system [37] and its accompanying software version 2.12.39 (Videometer A/S, Herlev, Denmark) was used for the acquisition of multispectral images in 18 different, non-uniformly distributed, wavelengths, ranging from Vis (405 nm) to short wave NIR (970 nm). The same software was used for the extraction of the informative area (region of interest, ROI). The mean reflectance spectra within the informative area (i.e., mean intensity of pixels), along with the corresponding standard deviation values for each image, were calculated and used for further analysis. In the present study, a similar approach to Panagou et al. [21] was applied for the data acquisition. In total, 272 images were collected and further analyzed.

### 2.4. Data Analysis

The aim of the present study was the assessment of the microbiological quality of chicken burgers in terms of three critical parameters of spoilage: initial population, storage time, and temperature. For this purpose, the samples were classified in three groups, defined as: satisfactory quality (class A): 4–7 log CFU/g (n = 60), acceptable quality (class B): 7–8 log CFU/g (n = 74), and unacceptable quality (class C): >8 log CFU/g (n = 140). The determination of classes was based mainly on microbiological meat spoilage criteria and similar studies in the literature [9,38,39]. Furthermore, the distribution of microbiological data (TVC values) was considered in a way that the samples in each class were of more or less equal number (balanced across classes) with small-scale sensory evaluation conducted in parallel to the microbiological analysis. The latter indicated that odor and appearance were acceptable when TVC was 7–8 log CFU/g. The consistency of the chicken burgers (24% of each burger consisted of breadcrumb, vegetables, herbs and other materials) contributed to maintaining the odor and appearance of the burgers, even when the microbial load was high. At higher temperatures (please refer to the Results section) the LAB population was higher compared to that of *Pseudomonas* spp., which dominated at lower temperatures, contributing to less unacceptable characteristics of the examined burgers. For the FTIR and MSI data, the plots (mean ± standard deviation) for all classes (A, B and C) considered are available in the Supplementary Materials (Figure S1). After data collection, data analysis was designed to incorporate the various conditions (e.g., fresh, and frozen state of the samples, different batches) simulating, as far as possible, real scenarios in the industry. It should be stressed that the product recipe was the same in our study since a real market product was used. In Supplementary Materials, Figure S2, data for all the batches derived from FTIR and MSI are presented, and it is shown that differences among the batches can be attributed to inter-batch variability. In the framework applied, stratified sampling was employed so that 75% to 25% of the dataset was used for model development (training) and external validation, respectively. The partitioning schema was selected so that the different batches, storage conditions (i.e., storage temperatures), and different years of purchase could be included in both the training set and the validation set of the models. The advantages of the partitioning schema were the random selection of the samples and the use of a common strategy for both sensors so that the results would be comparable. The parameters optimized and details of the model training phase of development are described below for each of the five classification approaches/algorithms used. Partial least squares discriminant analysis (PLSDA), support vector machine (SVM), random forest (RF), multinomial log-linear logistic regression (LR) and ordinal logistic regression (OLR) classification approaches/algorithms, were trained and tested (using external validation). The algorithms were implemented using R v4.0.3 [40], Rstudio v1.3.1093 [41]. The five algorithms, and the respective packages used, are briefly described below:

(i) ‘mixOmics’ [42] for PLSDA method [43], which is a linear classification model. It was employed for the reduction of dimensions and for the prediction of sample classes. In the current study the number of PLS components and the prediction distances (i.e., mahalanobis, centroids, maximum distance) were defined based on the classification error rate (leave-one-out cross-validation) of the training set.
(ii) ‘e1071’ [44] was used for SVM [45,46] and was employed with a radial basis function (RBF) or linear kernel. Grid search coupled with 10-fold cross-validation was performed for tuning the parameters of cost and gamma for RBF and the cost for linear kernel.
(iii) ‘e1071’ [44] was used for tuning the parameters (i.e., the number of trees to grow, the number of variables randomly sampled as candidates at each split) calculating the accuracy rate of 10-fold cross-validation for the training set and random forest [47] for implementing RF, which is an ensemble learning method for classification.
(iv) ‘pls’ package [48] was used for extracting the PLS latent variables for the dimensionality reduction of the FTIR data and, subsequently, the ‘nnet’ [49] package was used to fit LR on the transformed (towards lower dimensions—due to memory issues caused by the large number of dimensions, i.e., >934m number of wavenumbers) FTIR data and on the raw MSI data. The number of LVs selected was defined as the knee-point of the curve of the variance explained vs. number of LVs.
(v) ‘pls’ was applied on FTIR and MSI. The number of components was defined as the maximum number of components exhibiting at least 0.5% variance explained from the previous component. Subsequently, the MASS [49] package was used to fit OLR on the transformed FTIR and MSI data. The only difference between (iv) and (v) is that, in the second case, the Y-variable is handled as an ordinal categorical variable, defining the order of the three classes considered, i.e., in the continuum, “satisfactory”: 4–7 log CFU/g, “acceptable”: 7–8 log CFU/g, and “unacceptable”: >8 log CFU/g.

The package ‘caret’ was employed for the calculation of the performance metrics: accuracy, recall, precision, and F1-score for the test set (external validation).

(1) $$\mathrm{Accuracy}=\frac{\mathrm{Samples}\;\mathrm{correctly}\;\mathrm{predicted}}{\mathrm{All}\;\mathrm{samples}}$$

(2) $$\mathrm{Recall}=\frac{\mathrm{Samples}\;\mathrm{correctly}\;\mathrm{predicted}\;\mathrm{in}\;i\;\mathrm{class}}{\mathrm{All}\;\mathrm{samples}\;\mathrm{of}\;\mathrm{the}\;i\;\mathrm{class}}$$

(3) $$\mathrm{Precision}=\frac{\mathrm{Samples}\;\mathrm{correctly}\;\mathrm{predicted}\;\mathrm{in}\;i\;\mathrm{class}}{\mathrm{All}\;\mathrm{samples}\;\mathrm{predicted}\;\mathrm{as}\;i\;\mathrm{class}\;\left(\mathrm{correct}\;\mathrm{or}\;\mathrm{not}\right)}$$

(4) $$F1-\mathrm{score}=\frac{2*\left(\mathrm{Recall}*\mathrm{Precision}\right)}{\left(\mathrm{Recall}+\mathrm{Precision}\right)}$$

## 3. Results and Discussion

### 3.1. Microbiological Analysis and pH Measurements

**Figure 1:**
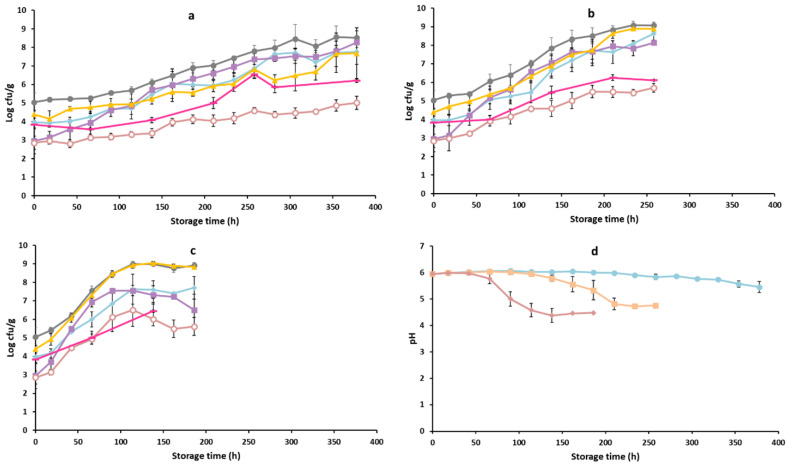
Populations (mean ± standard deviation, n = 4) of microbial groups of chicken burger during storage at 0 °C (**a**), 4 °C (**b**) and 8 °C (**c**) for batch 1 and 2; TVC (●), *B. thermosphacta* (■), *Pseudomonas* spp. (♦), LAB (▲), yeasts (–) and Enterobacteriaceae (o). pH values (**d**) of 0 °C (●), 4 °C (■) and 8 °C (♦).

The growth curves of the studied microbial groups of the examined commercial poultry product for b1 and b2 are presented in Figure 1.

The initial TVC was 5.04 (±0.51) log CFU/g and the pH was 5.95 (±0.05) for the chicken burgers (Figure 1). The microbial groups mainly contributing to the spoilage of chicken burgers under the conditions examined (0, 4, 8 °C) were LAB, *Pseudomonas* spp. and *B. thermosphacta*. As expected, the dominance of microbial groups varied among the examined temperatures. As illustrated in Figure 1, the initial population of *B. thermosphacta* (2.95 ± 0.71 log CFU/g) was relatively low, followed by *Pseudomonas* spp. (3.97 ± 0.06 log CFU/g) and LAB (4.38 ± 0.21 log CFU/g). The population of *B. thermosphacta* (8.27 ± 0.62 log CFU/g) was higher at the end of storage at 0 °C, compared to *Pseudomonas* spp. (7.76 ± 0.68 log CFU/g) and LAB (7.68 ± 1.38 log CFU/g). In contrast, for storage at 8 °C, LAB (8.83 ± 0.0.01 log CFU/g) reached a higher microbial population, followed by *Pseudomonas* spp. (7.71 ± 0.61 log CFU/g) and *B. thermosphacta* (6.51 ± 0.85 log CFU/g). The pH values at the end of storage at 0, 4, 8 °C were 5.45 ± 0.21, 4.75 ± 0.01 and 4.47 ± 0.01, respectively. The initial microbial populations of the Enterobacteriaceae family and yeasts were 2.85 ± 0.35 log CFU/g and 3.83 ± 0.22 log CFU/g, respectively, representing lower populations for all storage temperatures at the end of storage compared to the other microbial groups. The initial populations of the first two batches (year: 2018, Figure 1) were approximately 2 log CFU/g lower compared to the batches analyzed during year 2020 (Supplementary Material, Figure S3). The differences in microbial quality in terms of TVC may have been related to the different year of purchase (b1, b2: 2018 and b3–b6: 2020) and the different practices followed. For example, the chicken burgers in 2018 were provided fresh, but, in 2020, the product was only available frozen from the food industry. The differences among batches and between the two different years of purchase is of great importance, incorporating the variability occurring in real life. The main spoilage microorganisms were LAB, *B. thermosphacta,* and *Pseudomonas* spp. for samples provided during 2020. The microbial groups examined followed a similar growth pattern in 2018 and 2020. It is worth noting that, in a survey study which took place in Spain, the chicken burgers examined had a rather high population of 6.29 ± 0.64 log CFU/g, n = 5 [50]. The pH followed a similar pattern (comparing year 2018 with 2020) with a greater decrease at higher temperature (8 °C, 4.80 ± 0.58) compared to lower temperature (0 °C, 5.85 ± 0.11), which can be interpreted in terms of the development of the microbial groups and the higher population of LAB at higher temperatures (i.e., 8 °C).

As is indicated in the results of the microbiological analyses, the microbial groups contributing most to chicken burger spoilage were LAB, *Pseudomonas* spp. and *B. thermosphacta*. In similar studies, where intact or minced poultry meat has been studied, the main spoilage microorganisms were mainly *Pseudomonas* spp. for aerobic packaging conditions, or LAB and *B. thermosphacta* for vacuum and modified atmosphere packaging. In the case of high temperatures, Enterobacteriaceae probably contribute more to spoilage. It is well-known that the ingredients of the meat/poultry product, the packaging and the storage conditions strongly affect spoilage and the relative dominance of microorganisms [39,51,52,53].

### 3.2. FTIR Data

Table 1 presents the precision, recall, F1-score, and accuracy for the external validation using FTIR data and for all five algorithms considered.

In terms of accuracy scores, the best performance was achieved for classification when SVM (89.71%) was applied to FTIR data, followed by LR (88.24%), PLSDA (85.29%), RF (80.88%) and OLR (79.41%). A first observation in view of these results is that, while the OLR can theoretical be more efficient than LR, since it takes into account the order of the classes in the continuum (increasing microbiological loads on the samples), this is not the case here. A possible justification for this is that the order, as considered, is not supported by the data, at least, it is not reflected in the projected multidimensional space where the features are projected. So, while the class order is indicated by the TVC values, the classes do not follow that order in the variable space, i.e., class A may be closer to class C than class B (please refer to Table 1 where the efficiency of correctly identifying class B is severely downgraded in comparison to the LR approach). The A class corresponds to the satisfactory quality group of chicken burgers and was clearly discriminated (precision = recall = 100%) only when LR was applied to the FTIR data. LR and SVM showed similar performance in terms of accuracy (88.24% and 89.71%, respectively), but attaining 100% precision when LR was applied on FTIR data for class A is very important from the point of view of quality control, showing that none of the samples with lower quality were misclassified as being of ‘satisfactory quality’. The B class was discriminated less easily (F1-scores: 0.63–0.80) compared to the other classes, C (F1-scores: 0.84–0.93) and A (F1-scores: 0.87–1.00). It is worth noting that all misclassifications occurred between classes A and B or B and C classes, except in the case of RF, where samples from C class were misclassified as A. The latter case was a fail since it is considered a dangerous misclassification from both the consumer and industry points of view.

FTIR data have been used for detecting the number of days of storage after slaughter [29]. The evaluation of microbiological spoilage of chicken breast was also assessed by Vasconcelos et al. [9], attaining an RMSE prediction of 0.789 log CFU/g, while similar results were obtained by [38]. The above-mentioned studies examined the potential of FTIR data for the assessment of chicken breast fillets spoilage, in contrast to our case, where a chicken product with 76% meat was studied. Furthermore, it is also worth highlighting that a large number of samples were employed, acquired from different batches and from two different years, with the aim of developing more general and robust models, to help overcome underlying stochasticity inherent in the samples.

### 3.3. MSI Data

In Table 2, the respective values are presented for external validation using MSI data.

The performance, in terms of accuracy of scores, ranged from 74.63% (RF) to 85.07% (PLSDA). RF was the only case where a sample from the C class (unacceptable quality) was misclassified as A class (satisfactory quality). In all cases, samples belonging to the A class were classified correctly (recall = 100%), except in the case of SVM and OLR, where, in both cases, recall was 92.86%. The OLR and LR approaches applied on MSI data showed the same performance in terms of accuracy (82.09%). As discussed in the previously described case of FTIR, it was clear that the MSI data (due to their lower complexity and dimensionality than the FTIR data) better supported, but not adequately, the order of the considered classes in the continuum of microbiological load and in the multi-dimensional space projected; thus, OLR and LR exhibited similar performance. Similarly, for the F1-scores achieved when FTIR data were used, the B class was discriminated less easily (F1-scores: 0.38–0.69) compared to class C (F1-scores: 0.83–0.90) and class A (F1-scores: 0.85–0.93), but, in the case of MSI, the values were slightly lower. When SVM and LR were applied to the data, the precision for class A was higher (92.86 and 87.50%, respectively) compared to PLSDA, OLR and RF (<82.35%). The ability of a method not to misclassify the samples belonging to classes B and C as A, which is indicated by high precision scores, is important to ensure that samples assessed to be of satisfactory quality will not be of deteriorated quality. Multispectral imaging was investigated for the assessment of the microbiological quality of chicken fillets using key wavelengths in the regions 910–1700 nm and 400–1000 nm, respectively [12,54]. The studies described showed promising results for the prediction of TVC, but limitations, such as a small dataset and the lack of external validation, create the need for further investigation and data augmentation. Spyrelli et al. [31] studied different poultry products at-line using MSI, achieving promising results, and the results presented are comparable with those obtained here. Chicken burgers (n = 131, batches = 3) were evaluated (regression) for microbiological quality with RMSE = 0.285 for the prediction dataset, when time from slaughter was also predicted/estimated. It is worth noting that RMSE was in the range of 0.160–0.383 for the different poultry products (i.e., chicken breast, chicken thigh, chicken burger, chicken marinated souvlaki) for the prediction dataset. Vis/NIR spectroscopy has also been investigated for monitoring the quality assessment (i.e., L, a, b, pH, moisture, drip loss, expressible fluid, and salt-induced water gain) of chicken breast fillets [8].

Overall, the results are promising in terms of quality discrimination of the samples, and somewhat safety considered, using two different sensors coupled with application of machine learning methods. The SVM algorithm applied on MSI and FTIR data showed high performance compared with the different algorithms applied per sensor (MSI: 83.58%, FTIR: 89.71%). PLSDA showed similar performance, whether on FTIR or on MSI data (accuracy= 85.29 and 85.07%, respectively), but, compared to FTIR, the results, in terms of accuracy, were one of the two worst performances, while for MSI represented the best performance. Comparing the results from the test sets (external validation) for both sensors, it is indicative that, in terms of accuracy scores, FTIR (79.41–89.71%) performed better than MSI data (74.63–85.07%), which was also verified by the values of F1-score FTIR (Class A: 0.87–1.00, Class B: 0.63–0.80, Class C: 0.83–0.93) and MSI (Class A: 0.85–0.93, Class B: 0.38–0.67, Class C: 0.83–0.90). It is evident that the two extreme classes (A and C) exhibited the best performances for either set of sensor data, while the intermediate class B was the most “difficult” to identify. It is worth noting that, apart from the inherent stochasticity during data acquisition and among samples, we must also deal with the stochasticity of microbiological measurements per se. It is known that variability in the range of ±0.5 logCFU/g is common in these measurements, leading to increased uncertainty in the classification of samples. So, it is expected that samples at the “borders” between adjacent classes lie in a blurred uncertainty region, mainly affecting performance on the intermediate class B. The slightly inferior performance of MSI compared to FTIR data is probably related to the wavelength region, while the MSI data considers a larger region of the sample. The MSI data are acquired in the region of visible and short near infrared, while the FTIR data are acquired in the region of middle infrared (MIR). The region 1800–900 cm^−1^ is strongly related to the metabolic activity of microorganisms and has been characterized as the metabolic fingerprint region; as described in [13,17,29,36], the peaks in this region have been ascribed to amide I, II, III, amines, fat and moisture. On the other hand, the Vis/NIR (short region of NIR) region is associated more with respiratory pigments (i.e., myoglobin, oxymyoglobin, metmyoglobin) (Vis), and proteins and moisture content (NIR) [31]. It is worth noting that the different batches had slight differences in colour; for example, different colours of peppers were used in the different batches (red, yellow). All these variances were incorporated in the training phase of model development, embedding the related information, while the test set (external validation) was created by stratified sampling so as to have a similar distribution of samples from the different batches as in the training set. These differences are expected to affect the results of MSI (405–970 nm) more than FTIR (1800–900 cm^−1^) due to their spectral characteristics.

The present study presents a method that could provide rapid results when screening a great number of samples in a short time, at low cost and non-destructively. The advantages of these methods are clear, since corrective actions could be applied on time and the quality control could be performed on each sample of the food supply chain. Similar studies have been carried out for fish, vegetables, meat and fruits [18,24,55,56]. The results from all these studies support the use of these methods, but there are still some challenges to overcome. The challenge of transferring this technology to large scale and other types of foods mainly concerns the construction of large data lakes. In addition, changes in a step of the food supply chain (e.g., fresh vs. frozen) should be investigated if it affects the ‘performance’ of the method. Then, after collection of representative data, the use of different spectroscopic/imaging techniques should be investigated and the best fitted (i.e., robust models) machine learning methods for extracting the useful information should be investigated. Investigation of the most informative features, data fusion strategies and high-level technologies, in terms of image capture and spectral acquisition, could be key to the generalization of outcomes in the most effective way. FTIR and MSI/HSI have been investigated in a wider range of applications, including food safety [57,58].

## 4. Conclusions

FTIR and MSI data, coupled with machine learning methods, showed promising results for the microbiological quality assessment of chicken burgers, a product which is a mixture of ingredients, affecting obtained signals by introducing additional variability to that already existing due to meat-spoilage per se. The FTIR data showed slightly better performance in terms of accuracy scores when the different algorithms were applied compared to the performance of the MSI data. However, as indicated, further research is needed in terms of large-scale applicability and the development of general models to overcome the limitations of the product-, sensor- and algorithm-specific properties of these methods.

## Figures and Tables

**Table 1:** Precision (%), recall (%), F1-score, and accuracy (%) for PLSDA, SVM, RF, LR, and OLR classification models for external validation (n = 68) of chicken burger samples using FTIR data, considering three microbiological quality classes: A (satisfactory), B (acceptable), C (unacceptable).

		True Class		
		A	B	C
PLSDA	Precision (%)	88.24	75.00	88.57
	Recall (%)	100.00	66.67	88.57
	F1-score	0.94	0.71	0.89
	Accuracy (%)	85.29		
SVM	Precision (%)	88.24	82.35	94.12
	Recall (%)	100.00	77.78	91.43
	F1-score	0.94	0.80	0.93
	Accuracy (%)	89.71		
RF	Precision (%)	82.35	70.59	85.29
	Recall (%)	93.33	66.67	82.86
	F1-score	0.87	0.69	0.84
	Accuracy (%)	80.88		
LR	Precision (%)	100.00	77.78	88.57
	Recall (%)	100.00	77.78	88.57
	F1-score	1.00	0.78	0.89
	Accuracy (%)	88.24		
OLR	Precision (%)	87.50	60.00	87.50
	Recall (%)	93.33	66.67	80.00
	F1-score	0.90	0.63	0.84
	Accuracy (%)	79.41		

**Table 2:** Precision (%), recall (%), F1-score, and accuracy (%) for PLSDA, SVM, RF, LR, and OLR classification models for external validation (n = 67) of chicken burger samples using MSI data, considering three microbiological quality classes: A (satisfactory), B (acceptable), C (unacceptable).

		True Class		
		A	B	C
PLSDA	Precision (%)	82.35	78.57	88.89
	Recall (%)	100.00	61.11	91.43
	F1-score	0.90	0.69	0.90
	Accuracy (%)	85.07		
SVM	Precision (%)	92.86	73.33	84.21
	Recall (%)	92.86	61.11	91.43
	F1-score	0.93	0.67	0.88
	Accuracy (%)	83.58		
RF	Precision (%)	73.68	62.50	77.50
	Recall (%)	100.00	27.78	88.57
	F1-score	0.85	0.38	0.83
	Accuracy (%)	74.63		
LR	Precision (%)	87.50	66.67	87.88
	Recall (%)	100.00	66.67	82.86
	F1-score	0.93	0.67	0.85
	Accuracy (%)	82.09		
OLR	Precision (%)	81.25	71.43	86.49
	Recall (%)	92.86	55.56	91.43
	F1-score	0.86	0.62	0.89
	Accuracy (%)	82.09		

## Data Availability

The data presented in this study are available on request from the corresponding authors. The data are not publicly available due to privacy restrictions.

## Supplementary Materials

The following supporting information can be downloaded at: https://www.mdpi.com/article/10.3390/foods11162386/s1, Figure S1. Plots (mean ± standard deviation) of each class for MSI and FTIR data; class A (●), class B (●), and class C (●); Figure S2. Principal component analysis for all data from batches: b1, b2, b3, b4, b5 and b6 for FTIR and MSI data. Scores plots for PC1 and PC2; Figure S3. Populations (mean ± standard deviation) of microbial groups of chicken burger during storage at 0 °C (a), 4 °C (b) and 8 °C (c) for b4–b6; TVC (●), *B. thermosphacta* (■), *Pseudomonas* spp. (♦), LAB (▲), Yeasts (–) and Enterobacteriaceae (o). pH values (d) of 0 °C (●), 4 °C (■) and 8 °C (♦) (year: 2020).
