# Supplementary material for: Spectroscopic Data for the Rapid Assessment of Microbiological Quality of Chicken Burgers
Source: Foods. 2022 Aug 9;11(16):2386. doi: 10.3390/foods11162386 (PMC9407583; doi:10.3390/foods11162386)
Supplement: Supplementary file 1 [file foods-11-02386-s001.zip › foods-1820414-supplementary.pdf]

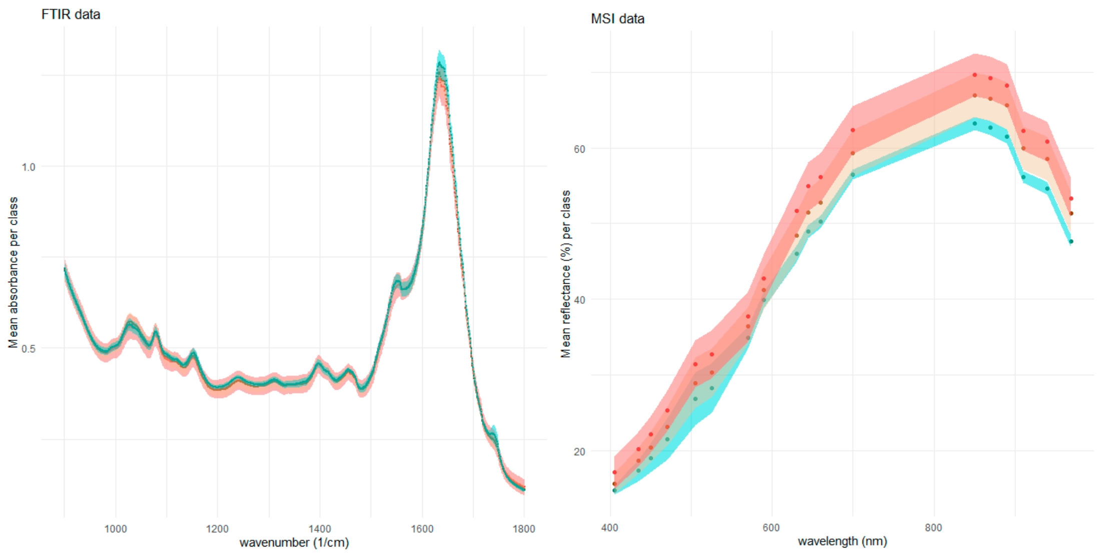

Figure S1. Plots (mean ± standard deviation) of each class for MSI and FTIR data; class A (●), class B (●), and class C (●).

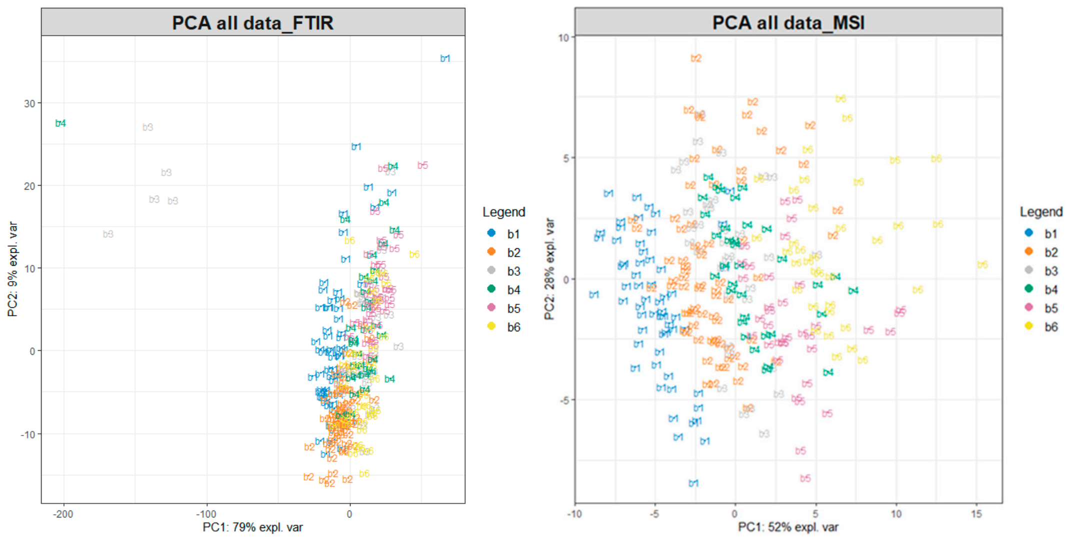

Figure S2. Principal component analysis for all data from batches: b1, b2, b3, b4, b5 and b6 for FTIR and MSI data. Scores plots for PC1 and PC2.

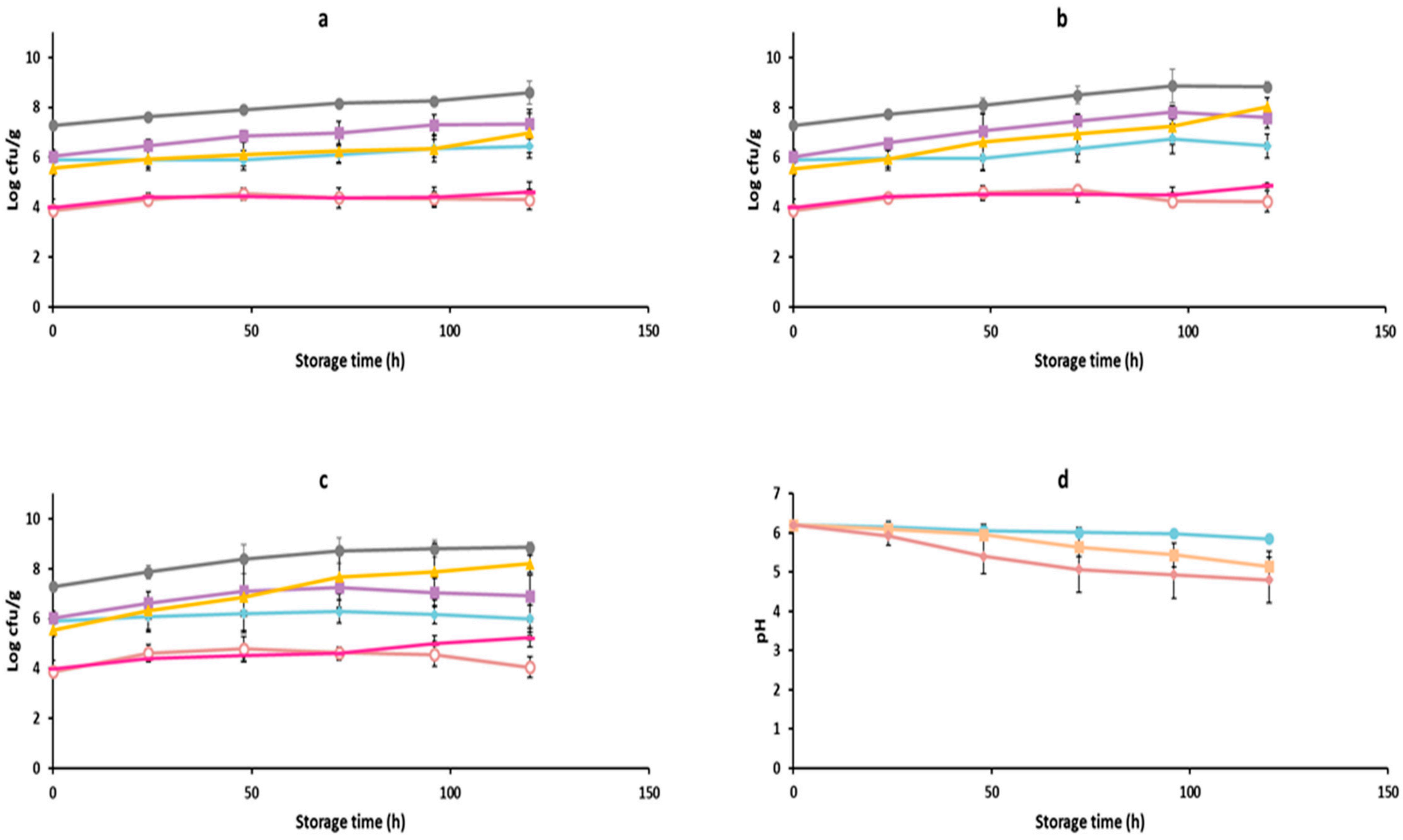

Figure S3. Populations (mean ± standard deviation) of microbial groups of chicken burger during storage at 0ºC (a), 4ºC (b) and 8ºC (c) for b4-b6; TVC (●), *B. thermosphacta* (■), *Pseudomonas* spp. (♦), LAB (▲), Yeasts (–) and Enterobacteriaceae (o). pH values (d) of 0ºC (●), 4ºC (■) and 8ºC (♦) (year: 2020).
